# Application of a multimedia-supported manikin system for preclinical dental training

**DOI:** 10.1186/s12909-022-03757-1

**Published:** 2022-09-27

**Authors:** Yi Yang, Gu Cheng, Xin Xing, Zhi Li, Wei Zhang

**Affiliations:** 1 The state key laboratory breeding base of basic science of stomatology (Hubei-MOST) & key lab for oral biomedical engineering of the ministry of Education, School & Hospital of Stomatology, Wuhan University, Wuhan, China; 2 West China Hospital of Stomatology, Sichuan University, Chengdu, China; 3 Department of Oral & Maxillofacial Surgery, School & Hospital of Stomatology, Wuhan University, Wuhan, China

**Keywords:** Manikin, Multimedia system, Preclinical training, Preclinical practice, Dental medicine

## Abstract

**Aim:**

In this study, we aimed to describe a multimedia-supported manikin system, compare the new manikin with the traditional manikin and evaluate its effectiveness in preclinical dentistry training.

**Methods:**

A total of 150 students participated in this study. Amongst these students, 71 in the 2015-year group used traditional manikins (Group TM) for preclinical training courses (endodontics training courses and prosthodontics training courses), and 79 in the 2016-year group used manikins with a multimedia system (Group MM). The scores of the training courses between the two groups were compared. A questionnaire survey was used to collect opinions of the students in Group MM on their experience of using the multimedia-supported manikin system in the preclinical training.

**Results:**

In the endodontics training courses, the scores of Group MM were higher than those of Group TM, but there was no significant difference (*P* = 0.379 > .05). However, the scores of prosthodontics training courses in Group MM were significantly higher than those in Group TM (*P* = 0.018 < .05). The questionnaire results indicated that the students in Group MM were satisfied with the device in usability, clarity, effectiveness and improvement in operation proficiency.

**Conclusions:**

In the groups studied, for preclinical dental training, the multimedia-supported manikin system was a good alternative to traditional manikin in preclinical dentistry training.

## Background

Dentistry, as a discipline with equal emphasis on theory and practise, is characterised by the irreproducibility and irreversibility of many clinical practices [1]. Therefore, preclinical training using manikins is necessary [2]. The application of manikins with artificial teeth to practise dental procedures continues to form the bulk of preclinical training in dentistry [3, 4]. These manikins are always placed in a laboratory or attached to a dental chair to create a convenient and safe environment for both students and physicians [5, 6]. This way easily allows educators to ensure that the desired learning goals are met [7]. To some extent, practicing as much as possible can reduce the chance of making mistakes [8].

However, the traditional manikins exist some shortcomings. Evidence for the validity of traditional manikins is limited [1]. When students operate on their own, it is not easy for the teacher to instruct students individually on posture and technique [9]. Moreover, due to the lack of complete manipulation equipment and simulation conditioning systems, preclinical training with traditional manikins does not fully simulate clinical situations [10].

We have witnessed rapid advancements in digital technologies for dentistry in recent decades [9]. In this study, we describe a new unified device: a multimedia-supported manikin system. It is a set of modern dental training equipment which can completely simulate the real clinical operating environment. The multimedia-supported manikin system integrates an advanced integrated dental treatment machine, a high-tech multimedia system with a high-fidelity manikin that mimics the patient’s real jaw, head, and neck. Multimedia enables users to navigate, interact, create and communicate [11]. The casters and an electric shoulder lift equipped on the multimedia-supported manikin system help the adaptation of postural adjustments for patients in clinical settings. The strong magnets between the dentition and mandible make ensure the correct position [12]. The integrated treatment system includes dental handpieces, a centralised air supply, water preset, gas path and foot switch control.

The digital interactive system mainly includes a digital microscope, recording device, a digital image processing and analysis system, an interactive application teaching software system, a high-speed video and audio acquisition system, a multimedia display system and a computer local area network (LAN) system. The digital interactive system has the functions of screen broadcasting, voice teaching, screen monitoring. Specifically, the teacher can adjust the teaching methods and contents based on the monitored situation. In this way, we strive to enable each student to master basic operations and reduce barriers to entry into the clinic.

The aim of this study was to describe a multimedia-supported manikin system, to compare the new manikin with the traditional manikin, and to evaluate its effectiveness in improving students’ practical skills. We also aimed to analyse the advantages and disadvantages of the new manikin and conduct a summative evaluation of students’ experience with the multimedia-supported manikin system in preclinical training.

## Methods

**Fig. 1:**
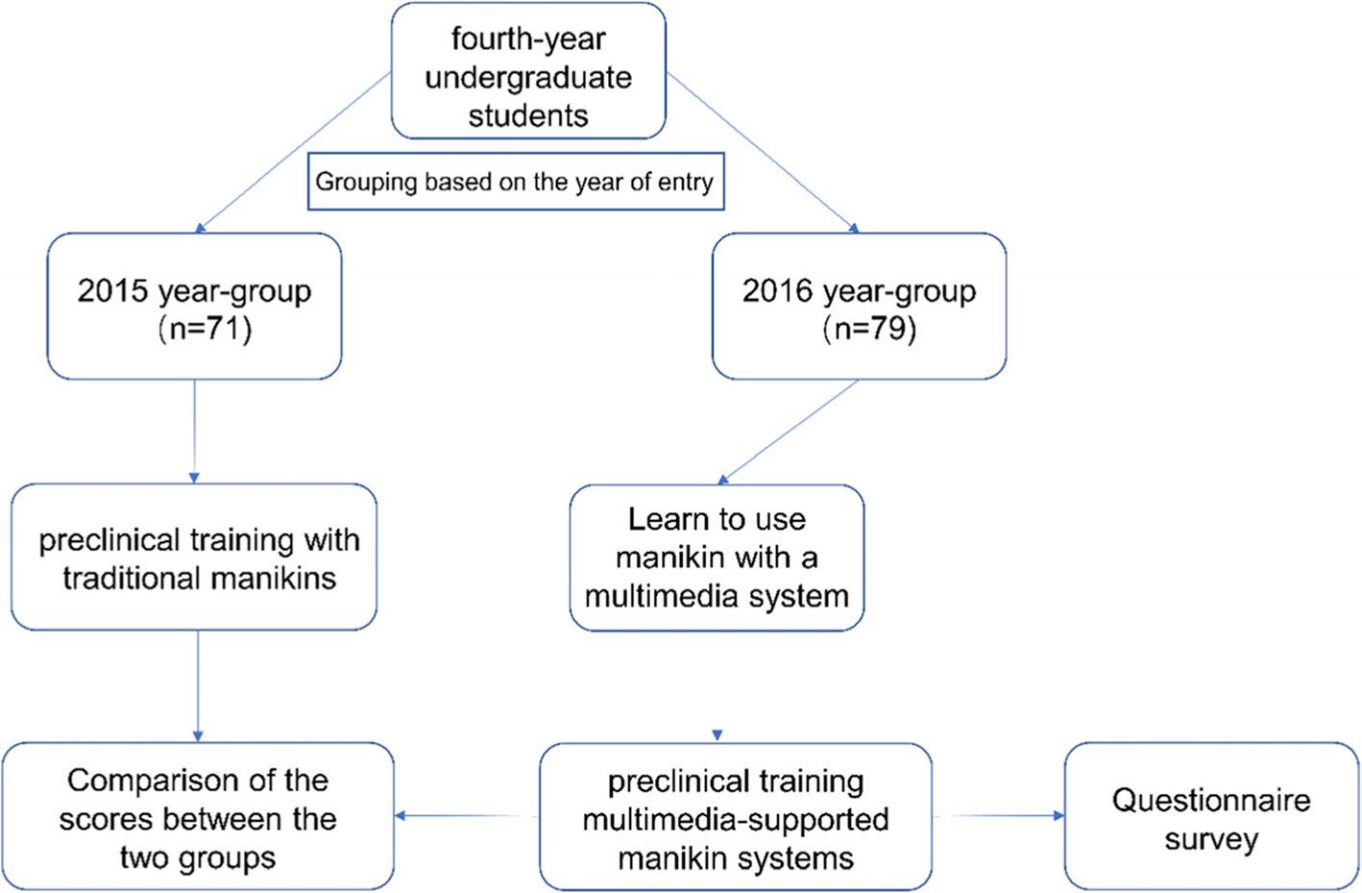
Flow chart

The eligible criteria for participants were the fourth-year undergraduate students of stomatology, who had taken theory courses in endodontics and prosthodontics. All eligible students admitted in 2015 and 2016 were recruited in our study and the grouping method was based on the year they entry. Specifically, 71 students (24 male, 47 female) admitted in 2015 took part in the study in 2018, while 79 students (32 male, 47 female) admitted in 2016 took part in the study in 2019. There was no significant difference (*P* = 0.397 > .05) between the two groups in male-female ratio according to Chi-square test. Amongst these students, 2015-year group used traditional manikins (Group TM) for the preclinical training and 2016-year group used multimedia-supported manikin systems (Group MM). The scores of the two groups were compared. Moreover, students in 2016-year group were required to complete questionnaire surveys anonymously after finishing training courses. The general flow of the whole study design was briefly depicted on Fig. 1. All participants involved in this study gave their informed consent. Institutional review board (IRB) approval of Ethics Committee of School & Hospital of Stomatology, Wuhan University was obtained for this study. The training topics discussed in the two groups were arranged in the same way and included common clinical endodontics and prosthodontics operations. Preclinical training tends to follow a carefully choreographed, lockstep program with paired lectures and laboratory sessions [13].

### Preclinical training on the traditional manikin

In traditional manikin-based preclinical training, the instructor first explained key points and then demonstrated the procedures on the manikin. The students observed the instructor in action. Since there were several students around, creating a limited field of view, the teacher needed to demonstrate several times. Then, the teacher instructed the students while the students practiced on the manikins. At the end of lessons, the teacher assessed the results of the students’ completed manipulations one by one.

### Training with the manikin with a multimedia system

This study required the ability of students and instructors to use the multimedia-supported manikin system. Therefore, all students in Group MM and instructors were trained in the use of the manikins prior to the preclinical training sessions to ensure teaching effectiveness.

The multimedia-supported manikin system can be used to teach a range of techniques from routine conservative procedures to complex endodontics involving microscopy [14]. Before the preclinical training courses, the functions of casters, elevation platform, integrated treatment system with high and low handpieces and suction devices, microscope, high and low handpiece frequencies, water and air circuits were learned.

**Fig. 2:**
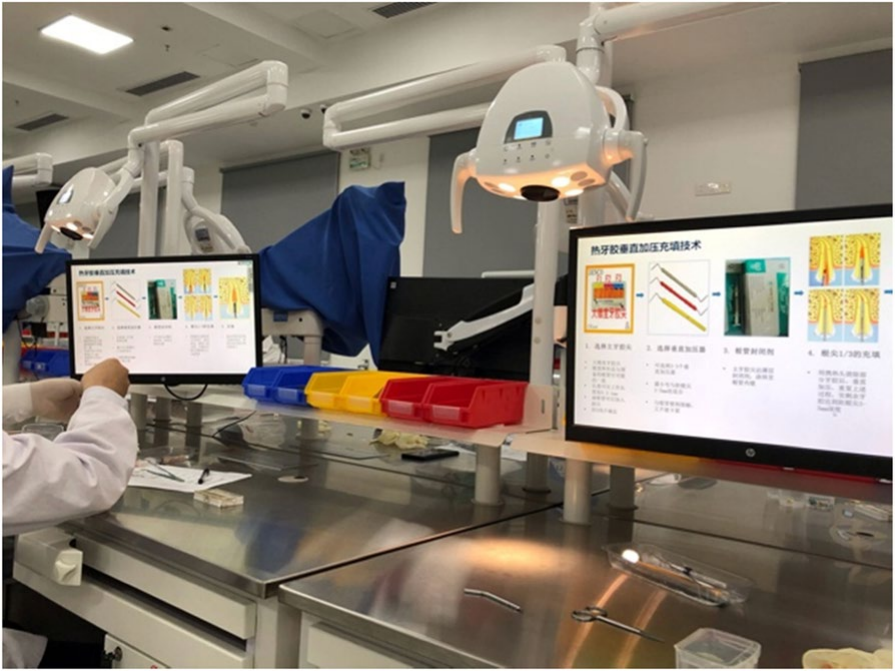
Student side displaying the teacher’s PPT

**Fig. 3:**
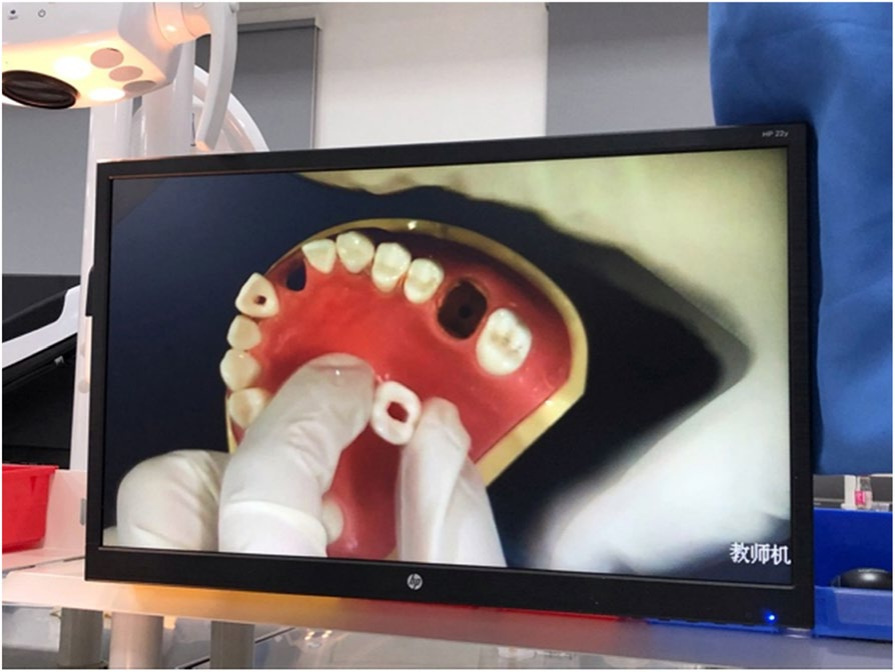
Teacher displaying the actions of a student

**Fig. 4:**
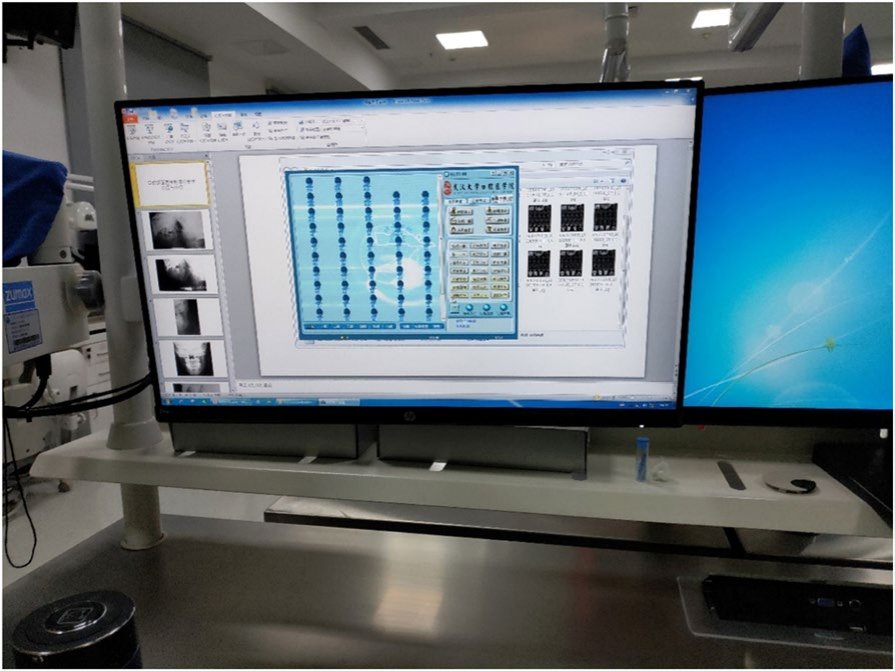
Interface of the student–teacher interaction system

The digital interactive teaching system consists of student operating, teacher operating and teacher–student interactive system. The student operating system can display all the contents of the teacher’s platform on a liquid crystal display screen for synchronise teaching (Fig. 2). Images of the students’ actions can be transmitted to the teacher operating system through the recording device (Fig. 3). Students can ask questions by clicking on the ‘hands up’ option through the teacher–student interaction system (Fig. 4). Depending on the teaching needs, we used an item bank provided by the system or loaded by the teacher to conduct synchronised web-based electronic examination.

### Preclinical training on the multimedia-supported manikin system

In the preclinical training courses, the instructor controlled the students’ operating system through the teacher’s operating system. The instructor then provided a theoretical explanation of the operation through a PowerPoint presentation or video. Next, the teacher performed on the manikin for demonstration. Each student’s display had a clear operating screen. Because of the clear view, most students operated independently after the teacher demonstrated once. Students who still had doubts about the operation could ask questions through the ‘hands up’ function of the system. Most students were still used to raising their hands directly to ask the teacher. The teacher always replied by walking up to the students or by providing a unified explanation publicly for frequently asked questions. And the teachers often chose to patrol the lab directly. At the end of each class, the teacher assessed the students’ results one by one after they have completed their manipulations.

### Comparison of the teaching effects between group TM and group MM

Preclinical training courses in endodontics and prosthodontics courses were very important for dental students. Therefore, in this study, we took these two preclinical training courses to compare the teaching effectiveness of the training methods based on the traditional manikin and the manikin with a multimedia system. The preclinical training courses in endodontics and prosthodontics lasted 48 hours, with a corresponding credit score of 1.5. These courses included instruction, independent practice, grading and examinations. Students in the TM and MM groups had the same duration of preclinical training courses.

### Questionnaire survey

The questionnaire consisted of seven items for the 79 students in Group MM to summarise the evaluation of their experience in preclinical practice. The seven items were provided to obtain information about the usability, clarity and effectiveness of the manikin with a multimedia system. Questions 1 and 2 correspond to usability, questions 3 and 4 to clarity, and questions 5, 6 and 7 to effectiveness. The objectives of this survey were to (1) evaluate the simulation degree of the multimedia-supported manikin system, (2) reflect the teaching effectiveness of the multimedia-supported manikin system and (3) assess the acceptance of the multimedia-supported manikin system. All students filled out questionnaire anonymously. A modified Likert scale that was designed and revised on the basis of previous studies and the objectives of the present study was used in the questionnaire [15–17]. The questionnaire provided five possible options to prompt a stated opinion rather than a neutral attitude.

The questionnaire assessed different dimensions of the manikin with a multimedia system: usability, clarity and effectiveness. In addition, we set a subjective question: advantages or disadvantages of the new manikins. After completing the questionnaire, we analysed the reliability and validity of the questionnaire. The Cronbach Alpha coefficient of the questionnaire was 0.819 (> 0.8). Therefore, the reliability of the questionnaire proved to be high. Then, we verified the validity of the questionnaire by calculating the KMO value. The KMO value was 0.751, ranging from 0.7 to 0.8. This indicated that the validity of the questionnaire was good.

### Statistical analysis

The results of the preclinical training courses scores were analysed by using the statistical software program SPSS version.24.0. After confirming the data distribution, the scores were compared using the student t-test. The data compared using the t-test were tested for homogeneity of variance. Student t-tests were conducted to compare differences of the results between the two groups. All data were expressed as mean (SD), and a significance level of 0.05 was used to test for statistical differences. Data from the questionnaire were also analysed by using SPSS version.24.0.

## Results

### Results of operation scoring

In the preclinical training courses in endodontics, the scores of group MM (*n* = 79) were higher than those of group TM (*n* = 71), but there was no statistically significant difference (*P* = 0.379 > .05). However, the scores for the preclinical training courses of prosthodontics in Group MM (*n* = 79) were significantly higher than those in Group TM (*n* = 71; *P* < .05; Table 1). The teachers scored in accordance with the examination standard of attending physicians.

**Table 1:** The scores for the preclinical training courses

Scoring results	Group TM (*n* = 71)	Group MM (*n* = 79)	t value	***P*** value
Endodontics	87.59 ± 1.679	93.65 ± 2.019	−19.834	.379**
Prosthodontics	80.49 ± 8.281	82.53 ± 9.674	−1.390	.018*

*P < .05. **P > .05

### Survey results

To ensure that we could extract valid information from the questionnaire, we examined the communality values of this questionnaire. The communality value was greater than 0.5, which proved that there were no unreasonable research items in the questionnaire (Table 2). The results also showed that the corresponding relationship between items and factors were basically consistent with our design. The students’ responses to these questions were shown in Table 3. The response rate of the questionnaire was 81.01% (64/79). The results showed that the multimedia-supported manikin system played a positive role in preclinical dentistry training. All students agreed that the multimedia-supported manikin system was safe to use (64/64, 100%). They also strongly agreed that the device could help them consolidate their theoretical knowledge (64/64, 100%). The majority of students believed that the multimedia-supported manikin system could help them improve learning proficiency (62/64, 96.88%). Many students also believed that the multimedia-supported manikin system helped them to master operations quickly (60/64, 93.75%) and adapt to the clinical practice life (60/64, 93.75%). The majority of students agreed that the multimedia-supported manikin system provided a realistic operating experience (53/64, 82.81%). More than half of the students thought that the presentation images were clear (39/64, 60.94%). The results also showed that the system needed further improvements especially in the clarity category.

**Table 2:** Results of communality (Factor 1: effectiveness; Factor 2: Usability; Factor 3: Clarity)

Question	Factor loading coefficient			Communality
	Factor 1	Factor 2	Factor 3	
1. The multimedia-supported manikin system is safe.	0.222	**0.920**	0.007	0.895
2. The multimedia-supported manikin system helps me master operations quickly.	0.318	**0.839**	0.178	0.836
3. The multimedia image is clear.	0.157	0.100	**0.970**	0.976
4. The multimedia-supported manikin system provides a realistic feel for operation.	**0.756**	0.152	0.220	0.643
5. The multimedia-supported manikin system helps me to consolidate my theoretical knowledge.	**0.842**	0.272	0.179	0.816
6. The multimedia-supported manikin system helps improve my learning proficiency.	**0.834**	0.348	0.059	0.820
7. The multimedia-supported manikin system helps me to adapt the clinical practice life.	**0.879**	0.187	0.002	0.808

**Table 3:** Questionnaire results

Questions		Strongly agree	Agree	Uncertain	Disagree	Strongly disagree
1. The multimedia-supported manikin system is safe.		41 (64.06%)	23 (35.94%)	0 (0.00)	0 (0.00)	0 (0.00)
2. The multimedia-supported manikin system helps me quickly master operations.		33 (51.56%)	27 (42.19%)	4 (6.25%)	0 (0.00)	0 (0.00)
3. The multimedia image is clear.		16(25.00%)	23 (35.94%)	14 (21.88%)	11 (17.19%)	0 (0.00)
4. The multimedia-supported manikin system provides a realistic feel for operation.		21 (32.81%)	32 (50.00%)	8(12.50%)	3 (4.69)	0 (0.00)
5. The multimedia-supported manikin system helps me to consolidate my theoretical knowledge.		33 (51.56%)	31 (48.44%)	0 (0.00)	3 (3.80%)	0 (0.00)
6. The multimedia-supported manikin system helps improve my learning proficiency.		33 (51.56%)	29(45.31%)	2(3.13%)	0 (0.00)	0 (0.00)
7. The multimedia-supported manikin system helps me to adapt the clinical practice life.		30 (46.88%)	30 (46.88%)	3 (4.69%)	1 (1.56%)	0 (0.00)

The responses ‘strongly agree’ and ‘agree’ were combined into a total agreement response

## Discussion

In the endodontics training courses, the scores of Group MM were higher than those of Group TM, but there was no significant difference (*P* = 0.379 > .05). However, the scores of prosthodontics training courses in Group MM were significantly higher than those in Group TM (*P* = 0.018 < .05). The comparative results of the scores between the two groups suggested that the multimedia-supported manikin system was helpful in improving the teaching effectiveness. The questionnaire results indicated that the students in Group MM were satisfied with the device in usability, clarity, effectiveness and improvement in operation proficiency, but needed further improvements especially in the clarity category.

Although the role of the clinical instructor is to ensure that students receive preclinical skills through different learning modalities, the role of advanced technologies are also very important [18]. Integrated devices compensated for the deficiencies of traditional manikins in teaching and manipulation. The operational scoring results of this study indicated that the use of a multimedia-supported manikin system was ultimately better than traditional manikins for teaching and learning. The results of the questionnaire showed that students were satisfied with the new device in usability, clarity and effectiveness. Almost all of the students’ free comments were positive. Some typical comments were as follows: ‘Easy to use and interactive.’ ‘It motivates me to acquire more knowledge.’ ‘It is fun and engaging.’ Including students’ perceptions in the educational process is considered a key component in monitoring the quality of academic programs [19]. The results indicated that in the groups studied, the multimedia-supported manikin system played a positive role in their preclinical dental training.

Traditional preclinical training courses were usually conducted by teachers based on textbooks and demonstrations. Since students were unable to see many manipulations in the mouth of the manikin, the teacher had to repeat the demonstrations. As a result, a considerable amount of time was spent on demonstrations. The multimedia-supported manikin system ensured that all the image data can be transferred from the teacher’s computer to students’ computer in real time. Taking advantage of its sophisticated teaching equipment and unique audio-visual advantages, the multimedia-supported manikin system combines instructional courseware, pictures, video materials and real-time demonstrations. These features deliver visual and auditory information to students, making abstract theory visual. This teaching method greatly enlivened the classroom atmosphere and increased the students’ interest in learning. In addition, students have an initial impression of the operation steps before watching the demonstration. The simulation system can completely simulate the clinical operation, which ensures the standardization of the teacher’s operation and teaching. During the preclinical training courses, students could carefully observe and imitate every precise and subtle demonstration, and form a concrete impression of the clinical operation in a limited time. As a result, most students begin practicing after a single demonstration by the instructor. When a difficult operation was encountered, students would ask for details of the operation. However, in general, the number of repetitions was reduced.

Students can even consolidate and review after the teacher’s presentation, as the multimedia system contains relevant E-learning materials, such as photos and videos. In addition to the teacher’s lessons, E-learning can also be used for efficient and diverse extended learning [20]. Well-designed E-learning instructional materials can help students consolidate their existing knowledge and competencies in important subjects and provide an enjoyable learning experience [21]. A great advantage of E-learning is that it frees interactions between learners and instructors or between learners and learners from constraints of time and space [22]. In multimedia systems, E-learning can increase the efficiency of self-learning and save instructor resources and costs.

In addition, the multimedia-supported manikin system has advantages in terms of communication between teachers and students. In Group TM, the teacher guided and corrected the students’ skills through inspection. In this traditional approach, it was not easy for the instructor to observe each student’s entire manipulation and provide immediate feedback. In addition, many operation errors were not corrected due to inefficient instructional guidance and students’ misunderstanding of the curriculum. The teaching method based on the multimedia-supported manikin system could solve the problem to some extent. Whilst students operated independently, the instructor could use the teacher’s operating system to capture the image on students’ recording devices and to observe the students’ operation. Students could also ask questions through the ‘hands up’ function of the software.

Third, in the approach of using the manikin with a multimedia system, students have fair chance and perspective to view demonstrations because each seat is equipped with the same clear images. In addition to the pedagogical advantages, multimedia-supported manikin systems also offer numerous operational improvements over traditional manikins. Simulation training is popular with students in medicine and dentistry [3, 23, 24] because it enables learners to develop clinical skills without fear of harming patients, helps retain didactic information and repeat critical skills, creates a positive learning experience and increases student motivation and satisfaction [25, 26]. The manikin used in this study is a type II homogeneous manikin system that included a simulated homogeneous jaw frame, face mask, skull cover and simulation shoulder that fully simulated the structure of the human body. These designs and configurations greatly meet the frequently expressed need of dental students for more realistic dental models and facilitate the transition of students from the classroom to the clinic [27].

Given the limitations of traditional manikins in terms of manipulation and teaching, students and teachers need new and improved teaching methods. Digital and multimedia teaching will definitely become a trend for the future [28]. In recent years, digital interactive systems have played an important and unique role in the educational process [29]. They have been used as a supplement to classroom presentations and laboratory teaching and have developed into a modern model of one-to-many education [30]. In-depth analyses of simulation studies in various branches of medicine have shown that high simulation can facilitate learning in the right environment [31]. With the progress of times and development of science and technology, we have reason to believe that this new teaching method will be further developed.

There are still some limitations in the practice methods based on multimedia-supported manikin systems. No technological development can yet simulate real patients. Moreover, the one-to-many teaching method based on LAN transmission is affected by the available network, so network delay will affect teaching quality. Although this method greatly widens the field of view, when complex operations are required, the camera position and angle are limited and students still cannot observe the instructor’s demonstration. In addition, the resolution of the computer image is inferior to that of human vision, and errors exist between the computer and microscope images. Some delicate operations and their results depend on human vision rather than the computer screen.

In addition, the manikin itself has shortcomings. Given that the manikin is only a representation of a person, some students are rude and do not take into account the feelings of the “patient”. This attitude hindered the achievement of satisfactory practice results. Since the manikin lacks the muscles and tissues of the lips and cheeks, it may not achieve the level of manipulation required by the clinic.

## Conclusions

The teaching effectiveness of the multimedia-supported manikin system was better in Group MM than Group TM. The effectiveness of preclinical training could be improved by the manikin with a multimedia system. Students in Group MM were satisfied with the multimedia-supported manikin system in effectiveness, usability and clarity and improvement in operation proficiency. In the groups studied, the manikin with a multimedia system was a good alternative to the traditional manikin in preclinical dental training.

## Data Availability

The datasets generated and/or analyzed in this study are not publicly available due to the sensitivity of the personal data of the study subjects as it relates to test scores. The datasets can only be available from the corresponding author on reasonable request.

## Abbreviations

TM: traditional manikins
MM: manikin with a multimedia system;
LAN: local area network
PPT: PowerPoint
